# A Prospective Observational Study: Are There Any Relationships Between Erythrocytosis, Renal Tubular–Glomerular Functions, and Systemic Hypertension in Adolescent Male Idiopathic Erythrocytosis Patients?

**DOI:** 10.3390/children13030427

**Published:** 2026-03-20

**Authors:** Nesrin Tas, Demet Baltu, Emel Ozyurek, Bulent Alioglu

**Affiliations:** 1 Department of Pediatric Nephrology, Ankara Training and Research Hospital, University of Health Sciences, Ankara 06010, Turkey; demetbaltu@gmail.com; 2 Department of Pediatric Hematology, Ankara Training and Research Hospital, University of Health Sciences, Ankara 06010, Turkey; emel.ozyurek@gmail.com (E.O.); alioglub@gmail.com (B.A.)

**Keywords:** hypertension, kidney, hematocrit, ambulatory blood pressure monitoring, adolescent

## Abstract

**Highlights:**

**What are the main findings?**

- Adolescent males with idiopathic erythrocytosis demonstrated higher ambulatory systolic and diastolic blood pressure load values when compared with healthy controls, despite similar mean blood pressure levels.
- Hematocrit levels were positively associated with several ambulatory blood pressure load parameters and remained independently associated with 24 h systolic blood pressure load in the multivariable regression analysis.

**What are the implications of the main findings?**

- Elevated hematocrit levels in adolescents with idiopathic erythrocytosis may contribute to early alterations in blood pressure regulation.
- These findings suggest that adolescents with idiopathic erythrocytosis may benefit from closer blood pressure monitoring to detect potential cardiovascular risk at an early stage.

**Abstract:**

**Background**: Childhood hypertension is an important predictor of adult cardiovascular disease. Idiopathic erythrocytosis in adolescent males is characterized by elevated hemoglobin and hematocrit levels, which may increase blood viscosity and potentially influence blood pressure (BP) regulation. However, the relationships between erythrocytosis, renal tubular–glomerular function, and systemic hypertension in adolescents remain unclear. **Methods**: This prospective observational case–control study was conducted between October of 2023 and April of 2024, including 37 male adolescents with idiopathic erythrocytosis and 24 age-matched healthy male controls. Complete blood count parameters were confirmed using two samples obtained at separate time points. Biochemical, urinalysis, tubular phosphorus reabsorption, and fractional excretion of sodium tests were performed to assess renal tubular and glomerular function, and 24 h ambulatory blood pressure monitoring (ABPM) was performed in all participants and interpreted according to the 2022 American Heart Association recommendations. **Results**: The mean systolic and diastolic BP values measured via ABPM did not differ significantly between the groups. However, adolescents with idiopathic erythrocytosis demonstrated significantly higher systolic and diastolic BP load values during 24 h, daytime, and nighttime periods when compared with healthy controls (*p* < 0.05). Renal tubular and glomerular function parameters were similar between groups. Hematocrit levels showed significant correlations with multiple ABPM load parameters. In the multivariable linear regression analysis, hematocrit remained independently associated with 24 h systolic BP load after adjustment for age, BMI, and serum creatinine. **Conclusions**: Adolescent males with idiopathic erythrocytosis exhibited increased ambulatory BP load despite similar mean BP values to controls. Elevated hematocrit may contribute to early alterations in BP regulation in adolescents with idiopathic erythrocytosis.

## 1. Introduction

Erythrocytosis is a condition in which the number of red blood cells, hemoglobin content, and hematocrit increase above their normal ranges (adjusted for age, sex, race, and altitude) [1,2]. Despite extensive testing, erythrocytosis remains unexplained in up to 70% of patients and is often labeled as idiopathic through a diagnosis of exclusion [2,3]. The increasing prevalence of hypertension and elevated blood pressure (BP) in childhood highlights the importance of identifying this condition in young individuals and addressing it through appropriate lifestyle changes and medications [4,5]. The cellular components of blood have a crucial impact on the blood’s viscosity, volume, and coagulability, which directly affect BP regulation [6,7]. Hematocrit is the most important factor affecting the viscosity of whole blood, and both blood viscosity and vascular resistance play roles in determining total peripheral resistance to blood flow [8].

There is still controversy surrounding the potential impacts of increased hematocrit levels and related increases in whole blood viscosity on the development of hypertension. In numerous clinical studies, a direct relationship has been demonstrated between BP and blood viscosity in both hypertensive and normotensive populations. Additionally, it has been reported that hypertensive individuals exhibit higher hematocrit levels [8,9,10,11]. On the other hand, some studies have failed to establish a conclusive link between high blood viscosity and hypertension, despite reports of changes in hemodynamic parameters, for example, increased blood vessel resistance due to changes in blood viscosity. However, according to published studies, these changes do not seem to have a significant effect on the cardiovascular system [12,13,14].

Therefore, this prospective study aimed to evaluate the relationships between erythrocytosis, renal tubular–glomerular functions, and systemic hypertension in adolescent male patients with idiopathic erythrocytosis.

## 2. Methods

This prospective observational case–control study was carried out at Ankara Training and Research Hospital from October 2023 to April 2024. The study was conducted according to the principles outlined in the Declaration of Helsinki and was approved by the Ethics Committee of Ankara Training and Research Hospital (approval no: 121). Written informed consent was obtained from all participants before they participated in the study.

A total of 37 male adolescents diagnosed with idiopathic erythrocytosis who were under evaluation at the hematology outpatient clinic and 24 age-matched healthy male controls were enrolled in this study. We only included male adolescents as idiopathic erythrocytosis is more frequently observed and clinically recognized in adolescent males in our clinical practice. In addition, restricting the study population to one sex helped to minimize potential confounding effects related to sex-related physiological differences in hemoglobin and hematocrit levels during adolescence. Participants were limited to adolescents older than 13 years of age, as the ambulatory blood pressure monitoring (ABPM) thresholds and classifications defined by the 2022 American Heart Association (AHA) guidelines are clearly applicable to this age group. None of the participants reported active cigarette smoking during the study period.

The control group consisted of age- and sex-matched healthy adolescents who attended the hospital for routine health examinations or minor non-chronic conditions. None of the control participants had a history of hematological, renal, or cardiovascular disease. Individuals with chronic illness, medication use affecting BP, or abnormal laboratory findings were excluded. Controls were recruited during the same study period as the erythrocytosis group in order to minimize potential selection bias.

Anthropometric measurements, including weight and height, were recorded for both groups. The body mass index (BMI) and standard deviation score (SDS) were then calculated using reference values for Turkish children [15]. The patient’s complete blood count (CBC) parameters were confirmed from two separate blood samples taken at different time points. In the idiopathic erythrocytosis group, all patients had normal erythropoietin (EPO) levels (at least 2 times) and tested negative for *JAK2* gene mutation. Abdominal ultrasound did not reveal any renal or hepatic lesions or signs of organomegaly.

In addition, all patients with erythrocytosis included in the study were found to have arterial oxygen saturation and arterial blood gases within normal ranges. Blood biochemical parameter and urinalysis tests were conducted, which involved detecting pH, density, and spot urine parameters. The tubular phosphorus reabsorption (TPR) and the fractional excretion of sodium (FENa) were analyzed according to the literature [16,17], in order to assess potential renal tubular and glomerular involvement that might contribute to BP regulation. In particular, FENa was used as an indicator of renal sodium handling, which is closely related to the pathophysiology of hypertension, whereas TPR was analyzed to evaluate proximal tubular function.

Office systolic and diastolic blood pressures were measured using the standard method. A Nihon Kohden bedside monitor (BSM-2301K) with an appropriately sized cuff was utilized to measure office BP.

Twenty-four-hour ABPM was performed on all patients using a PhysioPort long-term BP monitor (Model no: 302165, PAR Medizintechnik GmbH, Berlin, Germany). Children were instructed to wear the monitor on a typical day with an appropriately sized cuff on their non-dominant arm. Measurements were taken every 20 min during the day and every 30 min at night, with daytime was defined as 08:00 to 23:00 and nighttime as 23:00 to 08:00. Monitoring was considered adequate if the following criteria were met: at least 40–50 readings for a full 24 h report and 70% of all possible readings for a partial day report. The mean systolic blood pressure (SBP), diastolic blood pressure (DBP), and arterial pressure (MAP) levels and loads (percentage of readings above the ambulatory 95th percentile) were calculated.

Abnormalities were classified according to the AHA recommendations for children and adolescents, updated in 2022. All patients were at least 13 years of age. Ambulatory hypertension was determined if BP was equal to or exceeded 125/75 mmHg during 24 h, 130/80 mmHg during daytime, or 110/65 mmHg during nighttime and if office systolic and diastolic BP was equal to or exceeded 130/80 mmHg. Masked hypertension was determined if BP was equal to or exceeded 125/75 mmHg during 24 h, 130/80 mmHg during daytime, or 110/65 mmHg during nighttime and if office systolic and diastolic BP was below 130/80 mmHg. White coat hypertension was determined if BP was below 125/75 mmHg during 24 h, 130/80 mmHg during daytime, 110/65 mmHg during nighttime and if office systolic and diastolic BP were equal to or exceeded 130/80 mmHg. Normal BP was determined if BP was below 125/75 mmHg during 24 h, 130/80 mmHg during daytime, 110/65 mmHg during nighttime and if office systolic and diastolic BP were below 130/80 mmHg. Dipping refers to the percentage decline between day and night, calculated as [mean daytime BP—mean nighttime BP]/mean daytime BP. Non-dipping refers to a dipping level less than 10%, and reverse dipping refers to mean sleep BP exceeding wake BP [18,19].

### Statistical Analysis

The Kolmogorov–Smirnov test was used to determine the normality of the data. Descriptive parameters are expressed as mean ± standard deviation for normally distributed continuous variables and as median (interquartile range) for non-normally distributed continuous variables. Categorical parameters are expressed as numbers and percentages. The groups (idiopathic erythrocytosis vs. healthy control) were compared using independent sample *t*-tests, Mann–Whitney U tests, and chi-square tests. Variables with *p* < 0.05 were included in Spearman’s rank correlation coefficient analysis to determine whether there were any relationships between hemoglobin and hematocrit levels and the ambulatory BP monitoring results in the idiopathic erythrocytosis group. To investigate whether clinical and biochemical variables influenced BP load, multivariable linear regression analyses were performed for both 24 h systolic and diastolic BP loads. Age, body mass index (BMI), hematocrit, and serum creatinine levels were included in the models as potential confounders. Model assumptions were evaluated using residual histograms, normal P–P plots, and scatter plots of standardized residuals to assess normality and homoscedasticity.

Statistical analyses were performed using the Statistical Package for the Social Sciences for Windows, version 21.0 (IBM, SPSS Corp.; Armonk, NY, USA). Statistical significance was set at *p* < 0.05. A post hoc power analysis was performed using G*Power software (version 3.0, Germany). Assuming a two-sided independent sample *t*-test, an effect size of 0.8 (large effect according to Cohen), and a Type-I error (α) of 0.05, the calculated statistical power of the study was 85.1%.

## 3. Results

**Figure 1:**
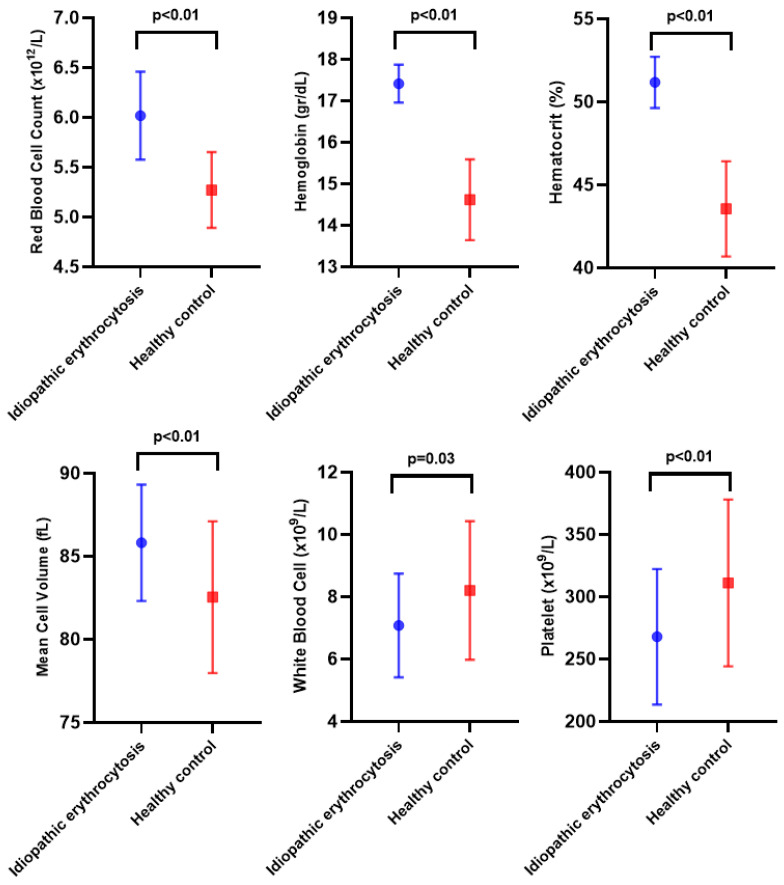
Comparison of complete blood count parameters between patients with idiopathic erythrocytosis and healthy controls.

A total of 37 patients with idiopathic erythrocytosis (15.9 ± 0.9 years) and 24 healthy controls (15.6 ± 0.9 years) were enrolled. No statistically significant difference in age was found between the groups (*p* = 0.16). The idiopathic erythrocytosis group had a BMI of 23.1 ± 3.4 kg/m^2^ and BMI-SDS of 0.2 ± 1.2, while the control group’s BMI was 24.2 ± 4.6 kg/m^2^ with BMI-SDS of 0.7 ± 1.5. No significant differences existed between the groups for BMI and BMI-SDS (*p*-values 0.28 and 0.12, respectively). A comparison of CBC parameters revealed that red blood cell count (6.0 ± 0.4 vs. 5.2 ± 0.4 × 10^12^/L), hemoglobin (17.4 ± 0.4 vs. 14.6 ± 0.9 g/dL), hematocrit [50.9 (1.9) vs. 44.3 (4.1)%], and mean corpuscular volume (85.8 ± 3.5 vs. 82.5 ± 4.5 fL) were higher in the idiopathic erythrocytosis group (*p*-values <0.01, <0.01, <0.01, and <0.01 respectively) (Table 1). Meanwhile, white blood cell count [6.6 (1.6) vs. 7.9 (2.1) × 10^9^/L] and platelet count (267.0 ± 54.0 vs. 311.0 ± 66.0 × 10^9^/L) were higher in the control group (*p*-values 0.03 and <0.01, respectively) (Table 1 and Figure 1).

No significant differences were observed between the idiopathic erythrocytosis and control groups for office SBP and DBP levels (*p*-values 0.12 and 0.22, respectively). The ABPM data showed no significant differences between the groups in mean SBP and DBP values across 24 h, daytime, and nighttime intervals (*p* > 0.05). The ABPM classification outcomes based on the AHA 2022 guidelines revealed no significant distinctions between the groups (*p* = 0.36). However, the idiopathic erythrocytosis group showed higher levels of 24 h SBP load [13.2 (21.8) vs. 7.7 (8.9)%], 24 h DBP load [25.0 (21.8) vs. 19.3 (15.5)%], daytime SBP load [10.0 (21.2) vs. 5.6 (7.1)%], daytime DBP load (22.8 ± 15.6 vs. 15.7 ± 9.2%), and nighttime SBP load [25.0 (37.1) vs. 12.5 (22.2)%], when compared to controls (*p*-values 0.01, 0.03, 0.02, 0.04, and 0.04, respectively). No significant differences were found between groups in mean arterial pressure, heart rate, SBP SDS, DBP SDS, MAP SDS, and HR SDS levels across 24 h, daytime, and nighttime intervals (*p* > 0.05), and the groups did not differ in terms of systole or diastole dipping patterns (*p* values 0.59 and 0.29, respectively) (Table 2).

Serum creatinine [0.82 (0.21) vs. 0.67 (0.15) mg/dL] and spot urine phosphate [25.1 (16.9) vs. 15.2 (14.9) mg/dL] levels were significantly higher in the idiopathic erythrocytosis group, compared to the healthy control group (*p*-values < 0.01 and 0.03, respectively). No other notable differences existed between the groups in serum and urinary biochemical parameters. Table 3 presents the analytical outcomes for both groups.

Spearman’s rank correlation coefficients showed no correlation between hemoglobin and ABPM loads in the idiopathic erythrocytosis group. However, there were significant relationships between blood hematocrit levels and 24 h SBP load, 24 h DBP load, daytime SBP load, daytime DBP load, and nighttime SBP load (*p* < 0.05). Spearman’s correlation coefficients are presented in Table 4.

Multivariable linear regression analysis was performed to evaluate independent predictors of 24 h BP load. For SBP load, the overall model was not statistically significant (R^2^ = 0.121, adjusted R^2^ = 0.059, F = 1.935, *p* = 0.117). Among the variables included in the model, hematocrit was independently associated with SBP load (β = 0.352, *p* = 0.012), whereas age, BMI, and serum creatinine were not significant predictors. None of the variables examined showed a significant independent association with DBP load.

## 4. Discussion

The flow resistance in a pipe is related to its geometric dimensions and fluid viscosity; as such, the two main factors determining vascular resistance are viscosity and vessel diameter [9]. In the pathophysiology of essential hypertension, increased blood viscosity and vascular resistance contribute to higher total peripheral resistance, consequently affecting blood flow.

Cinar et al. evaluated the effect of hematocrit on blood viscosity in healthy adults, and reported that a 10.99% increase in hematocrit caused a 20% increase in blood viscosity, reducing the blood flow rate by 16.67%. The researchers suggested that the use of vasodilators to increase blood flow could pose a risk of hypotension and stasis, noting that the compensatory increase in BP might cause extra circulatory workload and trigger hypertension [20]. Devereux et al. compared adult essential hypertension patients with normotensive controls and found that whole blood viscosity was 10% higher in hypertensive patients; however, they found no correlation between viscosity and hematocrit, noting that other mechanisms could contribute to the observed increase in blood viscosity. The authors concluded that elevated blood viscosity and resulting peripheral resistance contribute to high BP [9]. Letcher et al. studied 98 adults, including equal numbers of normal BP and untreated hypertension cases. They measured blood viscosity at six shear rates and found a significant correlation with BP levels; they also found that fibrinogen levels were increased in hypertensive patients independent of hematocrit values, indicating that plasma viscosity is influenced by fibrinogen [10]. Sileshi et al. reported a significant correlation between BP and hematocrit levels in 102 hypertensive and 102 healthy subjects, arguing that high hematocrit contributes to the pathogenesis of hypertension through increased blood viscosity [6]. Jeong et al. noted a connection between high hemoglobin and hematocrit levels and increased BP, interpreted through a different mechanism [21]. Nitric oxide, produced in endothelial cells, has a vasodilatory effect and protects against atherosclerosis-related complications [22]. They concluded that unbound hemoglobin scavenges nitric oxide, thus inducing vasoconstriction and contributing to hypertension [21].

In this study examining the relationship between erythrocytosis and hypertension in childhood erythrocytosis cases, no difference was found between patients with erythrocytosis and the control group regarding office systolic and diastolic blood pressures and the ABPM findings. However, the 24 h SBP and DBP, daytime SBP and DBP, and nighttime SBP loads were significantly higher in individuals with erythrocytosis than the control group, which was similar in terms of age, gender, and demographic characteristics. Our study revealed a significant correlation between high hematocrit levels and arterial BP, with increased hematocrit levels corresponding to higher 24 h SBP and DBP, daytime SBP and DBP, and nighttime SBP loads. The multivariable linear regression analysis indicated that hematocrit remained independently associated with systolic BP load after adjusting for age, BMI, and serum creatinine, while no independent predictors were identified for diastolic BP load. While hyperviscosity and secondary hypertension are common in erythrocytosis patients, understanding of hypertension in idiopathic erythrocytosis remains limited, with information primarily coming from publications on secondary erythrocytosis cases. Our study evaluated hemoglobin and hematocrit measurements but not viscosity, which is a limitation. Furthermore, although increased hematocrit increases whole blood viscosity, blood viscosity was not directly measured; therefore, the link between hematocrit and BP loads in our cohort should be interpreted cautiously. Future studies assessing direct hemorheological measurements will be necessary to clarify whether increased viscosity mediates this association.

Simone et al. found that whole blood viscosity was higher in men, smokers, and those with central obesity but was not associated with hypertension or diabetes [23]. Bogar concluded that there is no causal relationship between hemorheology and hypertension, as both conditions may arise from similar factors such as obesity, chronic mental stress, physical inactivity, and smoking [12]. The development of hypertension is influenced by genetics, neurogenic factors, sodium and water balance, hormones (e.g., renin, angiotensin, and aldosterone), metabolic abnormalities, oxidative stress, autonomic nervous system, local vascular factors, vasopressin, perinatal risk factors, and environmental factors [24]. As we did not assess blood viscosity, we can only speculate that the high BP loads in the erythrocytosis group might have been caused by increased blood viscosity.

Although our investigation revealed a connection between hematocrit levels and BP loads, the 2022 AHA guidelines state that ambulatory load measurements can be disregarded for diagnosing hypertension [19]. Hamdani et al. showed that, in healthy adolescents and adults, BP load had no independent connection to left ventricular hypertrophy (LVH), and including BP load with mean BP levels did not improve LVH prediction [25]; however, Gupta et al. found that 17.4% of patients had unclassified hypertension, with normal casual and ambulatory BP but elevated BP loads, which was linked to LVH [26]. Samuels et al. considered BP load elevations as indicative of hypertension in chronic kidney disease patients [27]. The impact of isolated BP load elevation remains unclear, and the AHA guidelines do not address how to categorize ABPM results showing elevated loads but normal average BP. Given the high loads observed in this study, we conclude that such loads are important in the idiopathic erythrocytosis patient group, requiring close monitoring for the development of hypertension in adulthood.

One possible mechanism underlying hypertension may involve alterations in renal sodium handling. Sodium is the principal cation found outside cells and is considered the most crucial environmental factor driving this condition [28]. Impaired handling of sodium in the kidneys contributes to the development of hypertension, being associated with salt-sensitive high BP [29]. Renal handling of sodium is best evaluated with the FENa. Our research examined kidney tubular and glomerular functions potentially involved in the development of hypertension in erythrocytosis patients, which has not been addressed in previous studies. Our findings revealed no kidney tubular or glomerular dysfunction in idiopathic erythrocytosis cases—particularly regarding FENa—with results comparable to those in the healthy control group.

This study has limitations, including the absence of blood viscosity measurements, low number of patients, and short study period. Further studies with extended follow-up periods and diverse patient populations are needed to elucidate the long-term effects of high hematocrit on the etiology of hypertension. Furthermore, this was a single-center study. Methodologically, the consistency between group comparisons, correlation analyses, and the multivariable regression findings strengthens the robustness of the observed association between hematocrit and systolic BP load. Nevertheless, given the relatively small sample size and the exploratory nature of the regression models, these findings should be interpreted cautiously and confirmed in larger prospective cohorts. Another limitation of this study is that the study population consisted exclusively of male adolescents and did not include female participants or adolescent smokers. Therefore, the findings may not be directly generalizable to female patients with erythrocytosis.

In conclusion, this study revealed that ABPM loads were elevated in the idiopathic erythrocytosis group compared to healthy controls. Furthermore, a correlation between hematocrit and ABPM loads was observed, and hematocrit was independently associated with systolic BP load in the multivariable regression analysis. Further research with extended follow-up for idiopathic erythrocytosis cases is recommended, especially considering blood viscosity. Additionally, examining the structural components the contribute to peripheral resistance in hypertension is crucial, as well as determining whether hematocrit influences altered peripheral resistance or indicates the circulating blood volume. These research avenues may lead to enhanced strategies for the management and prevention of hypertension.

## Figures and Tables

**Table 1:** Blood parameters for the patients with idiopathic erythrocytosis and healthy controls.

*Characteristic*	Idiopathic Erythrocytosis (n = 37)	Healthy Control (n = 24)	*p* *
RBC (×10^12^/L)	6.0 ± 0.4	5.2 ± 0.4	**<0.01**
Hb (g/dL)	17.4 ± 0.8	14.6 ± 0.9	**<0.01**
Htc (%)	50.9 (1.9)	44.3 (4.1)	**<0.01**
MCV (fL)	85.8 ± 3.5	82.5 ± 4.5	**<0.01**
WBC (×10^9^/L)	6.6 (1.6)	7.9 (2.1)	**0.03**
Plt (×10^9^/L)	267.0 ± 54.0	311.0 ± 66.0	**<0.01**

Data are presented as the mean ± standard deviation or median (interquartile range). The bold numbers indicate *p* < 0.05. Abbreviations: Hb, hemoglobin; Htc, hematocrit; MCV, mean cell volume; WBC, white blood cell; Plt, platelet. * Independent sample *t*-tests and Mann–Whitney *U* tests were used to compare groups.

**Table 2:** Ambulatory blood pressure monitoring results and office blood pressures for patients with idiopathic erythrocytosis and healthy controls.

*Characteristic*	Idiopathic Erythrocytosis (n = 37)	Healthy Control (n = 24)	*p* *
Office SBP (mmHg)	128.7 ± 13.9	123.4 ± 11.3	0.12
Office DBP (mmHg)	76.6 ± 9.8	73.3 ± 11.0	0.22
***24 h***			
SBP (mmHg)	113.6 ± 8.1	111.3 ± 6.9	0.26
SBP load (%)	13.2 (21.8)	7.7 (8.9)	**0.01**
DBP (mmHg)	72.1 ± 6.5	70.2 ± 4.8	0.23
DBP load (%)	25.0 (21.8)	19.3 (15.5)	**0.03**
MAP (mmHg)	85.2 ± 6.7	83.2 ± 5.1	0.22
HR (bpm)	80.4 ± 8.2	82.3 ± 7.8	0.36
SBP SDS	−0.74 ± 1.0	−0.74 ± 1.0	0.99
DBP SDS	0.68 ± 1.1	0.43 ± 0.8	0.34
MAP SDS	0.1 ± 1.0	−0.1 ± 0.8	0.49
HR SDS	0.4 ± 1.0	0.5 ± 0.8	0.77
***Daytime***			
SBP (mmHg)	115.7 ± 8.7	112.8 ± 7.1	0.17
SBP load (%)	10.0 (21.2)	5.6 (7.1)	**0.02**
DBP (mmHg)	74.4 ± 7.5	72.1 ± 4.4	0.17
DBP load (%)	22.8 ± 15.6	15.7 ± 9.2	**0.04**
MAP (mmHg)	87.3 ± 7.7	85.2 ± 5.2	0.23
HR (bpm)	83.6 ± 8.7	85.3 ± 8.6	0.48
SBP SDS	−1.0 ± 1.1	−1.1 ± 1.1	0.81
DBP SDS	0.3 ± 1.2	−0.1 ± 0.8	0.21
MAP SDS	−0.1 ± 1.0	−0.3 ± 0.8	0.46
HR SDS	−0.1 ± 1.0	−0.1 ± 0.8	0.83
***Nighttime***			
SBP (mmHg)	106.8 ± 8.4	106.2 ± 10.3	0.78
SBP load (%)	25.0 (37.1)	12.5 (22.2)	**0.04**
DBP (mmHg)	64.9 ± 6.5	64.2 ± 7.8	0.73
DBP load (%)	37.5 (32.9)	25.0 (23.9)	0.08
MAP (mmHg)	78.2 ± 6.3	77.0 ± 7.7	0.53
HR (bpm)	69.9 ± 12.3	73.1 ± 9.4	0.28
SBP SDS	−0.04 ± 1.0	0.1 ± 1.2	0.70
DBP SDS	1.4 ± 1.1	1.3 ± 1.3	0.64
MAP SDS	0.7 ± 1.2	0.7 ± 1.2	0.84
HR SDS	0.5 ± 1.3	0.7 ± 1.4	0.46
***Dipping pattern***			
Systole			
Dipper	16 (26.2)	8 (13.1)	0.59
Non-dipper	21 (34.4)	16 (26.2)	
Diastole			
Dipper	24 (39.3)	12 (19.7)	0.29
Non-dipper	13 (21.3)	12 (19.7)	
***ABPM classification***			
Normal	14 (37.8)	12 (50)	
White Coat Hypertension	4 (10.8)	5 (20.8)	
Masked Hypertension	5 (13.5)	2 (8.3)	0.36
Ambulatory Hypertension	14 (37.8)	5 (20.8)	

Data are presented as mean ± standard deviation, median (interquartile range), or n (%). The bold numbers indicate *p* < 0.05. Abbreviations: ABPM, ambulatory blood pressure monitoring; DBP, diastolic blood pressure; HR, heart rate; MAP, mean arterial pressure; SBP, systolic blood pressure; SDS, standard deviation score. * Independent sample *t*-tests, Mann–Whitney *U* tests, and chi-squared tests were used to compare groups.

**Table 3:** Serum and urinary biochemical test results for the patients with idiopathic erythrocytosis and healthy controls.

*Characteristic*	Idiopathic Erythrocytosis (n = 37)	Healthy Control (n = 24)	*p* *
Urea (mg/dL)	23.7 ± 5.3	22.5 ± 5.6	0.39
Creatinine (mg/dL)	0.82 (0.21)	0.67 (0.15)	**<0.01**
Sodium (mEq/L)	142.0 (2.0)	141.0 (4.5)	0.28
Potassium (mEq/L)	4.4 ± 0.3	4.4 ± 0.4	0.53
Calcium (mg/dL)	10.1 (0.7)	9.8 (0.4)	0.10
Phosphate (mEq/L)	4.2 ± 0.4	4.4 ± 0.7	0.14
Urinary pH	7.41 (0.04)	7.35 (0.06)	0.99
Urinary density	1022.0 (9.0)	1024.0 (9.2)	0.27
Spot urine sodium (mmol/L)	126.4 ± 61.9	143.5 ± 64.2	0.30
Spot urine potassium (mmol/L)	61.6 ± 28.2	74.3 ± 15.3	0.05
Spot urine calcium (mg/dL)	8.7 (13.4)	7.8 (7.4)	0.56
Spot urine phosphate (mmol/L)	25.1 (16.9)	15.2 (14.9)	**0.03**
Spot urine protein/creatinine ratio (mg/mg)	0.09 (0.03)	0.1 (0.04)	0.67
Spot urine albumin/creatinine ratio (mg/g)	3.2 (3.0)	4.2 (3.7)	0.50
Spot urine calcium/creatinine ratio (mg/mg)	0.06 ± 0.03	0.05 ± 0.03	0.43
TPR (%)	97.6 (1.4)	97.7 (1.0)	0.46
FENa (%)	0.65 (0.58)	0.47 (0.24)	0.20
Spot urine β_2_-microglobulin, (mg/dL)	0.17 (0.04)	0.17 (0.01)	0.61

Data are presented as the mean ± standard deviation or median (interquartile range). The bold numbers indicate *p* < 0.05. Abbreviations: FENa, fractional excretion of sodium; TPR, tubular phosphorus reabsorption. * Independent sample *t*-tests and Mann–Whitney *U* tests were used to compare groups.

**Table 4:** Correlations between blood hemoglobin, hematocrit levels, and ambulatory blood pressure monitoring results in the idiopathic erythrocytosis group (n = 37).

	24 h SBP Load (%)	24 h DBP Load (%)	Daytime SBP Load (%)	Daytime DBP Load (%)	Nighttime SBP Load (%)
Blood Hb level (g/dL)					
Correlation coefficient	–0.007	0.11	−0.06	−0.02	−0.15
*p*-value *	0.66	0.94	0.71	0.86	0.36
Blood Htc level (%)					
Correlation coefficient	0.30	0.33	0.28	0.29	0.26
*p*-value *	**0.02**	**0.01**	**0.03**	**0.02**	**0.04**

The bold numbers indicate *p* < 0.05. Abbreviations: DBP, diastolic blood pressure; Hb, hemoglobin; Htc, hematocrit; SBP, systolic blood pressure. * Spearman’s rank correlation test was used to analyze the correlation.

## Data Availability

The original contributions presented in this study are included in the article. Further inquiries can be directed to the corresponding author.
